# Editorial on the Research Topic of Sports Training and the Promotion of Physical Health

**DOI:** 10.3390/ijerph20032663

**Published:** 2023-02-01

**Authors:** Markus Tannheimer

**Affiliations:** 1 Department of Sport and Rehabilitation Medicine, University of Ulm, Leimgrubenweg 14, 89075 Ulm, Germany; m.tannheimer@adk-gmbh.de; 2 Department of General and Visceral Surgery, Krankenhaus Blaubeuren, Ulmer Straße 26, 89143 Blaubeuren, Germany

## 1. Introduction

Sport activities are a deciding factor in maintaining or achieving physical health. Currently in focus are the effects of obesity or physical inactivity and the resulting diseases, such as type II diabetes and cardiovascular disease but also cancer, dementia, and depression [1,2]. Sport is a significant factor in the prevention and treatment of obesity [3], and there are efforts to re-establish sports among children [4]. These diseases cause high societal costs due to expensive medical therapies and the loss of productivity due to an inability to work [5,6,7]. Sport is effective in the therapy of type II diabetes and may even be superior to drug therapy [8].

However, sport can also be an important cause of injuries or overuse syndromes [9]. Training methods have a very significant influence on this. The goal of training in recreational sports is about far more than just increasing physical performance; it is at least as much about strengthening resilience to injury and being fun as well as enjoyable, i.e., it also has a positive influence on the psyche. Although it is indisputable that leisure-time exercise is generally associated with health benefits, it is a fact that dynamic sports in particular bear an inherent risk of injury due to accidents or interactions with opponents in team sports [10]. In elite sports injuries and illnesses often play a decisive role in whether an athlete is successful in the long term or not.

## 2. Examples for Research Ideas

In this way, we would like to show the wide range of health-promoting compensatory and therapeutic sports, via meritocratic amateur sports to elite sports, and focus on how the health of an athlete is best promoted or, in cases of elite athletes, is impaired as little as possible.

The need to develop training methodologies for sport as a therapeutic option for patients with, e.g., metabolic syndrome is essential to make this therapeutic approach attractive for them [11]. Research will also have to counteract the susceptibility of athletes to injuries due to constantly intensified training and increasingly high performance requirements in elite sports [12].

## 3. Key Focus of Contributions

The above being the case, this Special Issue, “Sports Training and the Promotion of Physical Health”, will not only include observational findings but will particularly focus on interventional studies tackling those serious challenges due to the increase in diseases caused by overnutrition or physical inactivity.

This Special Issue seeks all kinds of research papers on training management, active regeneration, and medical procedures for prevention, therapy, or rehabilitation to improve or restore the health of patients, the general population, and athletes, focusing on any risk factors, underlying causes and mechanisms, outcomes of, and/or suggestions for prevention studies. The spectrum of possible approaches and improvements is extremely diverse and ranges from basic research to individual athlete counseling and the therapy of patients in daily practice. We would like to encourage colleagues to submit any interdisciplinary work and multicountry collaborative research. We welcome all kinds of original research papers as well as systematic reviews and meta-analyses.

## References

[1] Collaborators G.O. (2017). Health effects of overweight and obesity in 195 countries over 25 years. N. Engl. J. Med..

[2] Blair S.N. (2009). Physical inactivity: The biggest public health problem of the 21st century. Br. J. Sports Med..

[3] Zwiauer K.F. (2000). Prevention and treatment of overweight and obesity in children and adolescents. Eur. J. Pediatr..

[4] Hills A.P., Dengel D.R., Lubans D.R. (2015). Supporting public health priorities: Recommendations for physical education and physical activity promotion in schools. Prog. Cardiovasc. Dis..

[5] Association A.D. (2008). Economic costs of diabetes in the US in 2007. Diabetes Care.

[6] Sortsoe C., Green A., Jensen P.B., Emneus M. (2016). Societal costs of diabetes mellitus in Denmark. Diabetic. Med..

[7] Moucheraud C., Lenz C., Latkovic M., Wirtz V.J. (2019). The costs of diabetes treatment in low-and middle-income countries: A systematic review. BMJ Global Health.

[8] Baptista L.C., Machado-Rodrigues A.M., Martins R.A. (2017). Exercise but not metformin improves health-related quality of life and mood states in older adults with type 2 diabetes. Eur. J. Sport Sci..

[9] Aaltonen S., Karjalainen H., Heinonen A., Parkkari J., Kujala U.M. (2007). Prevention of sports injuries: Systematic review of randomized controlled trials. Arch. Intern. Med..

[10] Junge A., Langevoort G., Pipe A., Peytavin A., Wong F., Mountjoy M., Beltrami G., Terrell R., Holzgraefe M., Charles R. (2006). Injuries in team sport tournaments during the 2004 Olympic Games. Am. J. Sports Med..

[11] Faria F., Howe C., Faria R., Andaki A., Marins J.C., Amorim P.R. (2020). Impact of recreational sports activities on metabolic syndrome components in adolescents. Int. J. Environ. Res. Public Health.

[12] West S.W., Clubb J., Torres-Ronda L., Howells D., Leng E., Vescovi J.D., Carmody S., Posthumus M., Dalen-Lorentsen T., Windt J. (2021). More than a metric: How training load is used in elite sport for athlete management. Int. J. Sports Med..

